# Characterizing the gut microbiota in females with infertility and preliminary results of a water-soluble dietary fiber intervention study

**DOI:** 10.3164/jcbn.20-53

**Published:** 2020-06-05

**Authors:** Shinnosuke Komiya, Yuji Naito, Hidetaka Okada, Yoshiyuki Matsuo, Kiichi Hirota, Tomohisa Takagi, Katsura Mizushima, Ryo Inoue, Aya Abe, Yoshiharu Morimoto

**Affiliations:** 1 HORAC Grand Front Osaka Clinic, 15th-floor tower B Grand Front Osaka, 3-1 Ofuka-cho, Kita-ku, Osaka 530-0011, Japan; 2 Obstetrics and Gynecology, Kansai Medical University Graduated School, 2-5-1 Shinmachi, Hirakata, Osaka 573-1010, Japan; 3 Molecular Gastroenterology and Hepatology, Graduate School of Medical Science, Kyoto Prefectural University of Medicine, Kyoto 602-8566, Japan; 4 Department of Human Stress Response Science, Institute of Biomedical Science, Kansai Medical University, 2-5-1 Shinmachi, Hirakata, Osaka 573-1010, Japan; 5 Department for Medical Innovation and Translational Medical Science, Graduate School of Medical Science, Kyoto Prefectural University of Medicine, Kyoto 602-8566, Japan; 6 Laboratory of Animal Science, Setsunan University, Nagaotoge-cho 45-1, Hirakata, Osaka 573-0101, Japan; 7 Nutrition Division, Taiyo Kagaku Co., Ltd., 1-3 Takaramachi, Yokkaichi, Mie 510-0844, Japan

**Keywords:** *Bifidobacterium*, dietary fiber, gut microbiota, infertility

## Abstract

Despite the advances in assisted reproductive technology, approximately 8–12% of the individuals worldwide who are willing to conceive are unable to do so. Fertility depends on a receptive state of the endometrium and hormonal adaptations as well as the immune system. Local and systemic immunities are greatly influenced by the microbiota. The aim of the present study was to compare the gut microbiota in female patients with that in infertility with fertile control subjects and to evaluate the effect of prebiotic partially hydrolyzed guar gum supplementation on gut dysbiosis and the outcome of pregnancy in patients treated with assisted reproductive technology. Dietary fiber can reconstitute the host intestinal microbiota and modify the immune function; however, clinical data regarding the effect of dietary fiber treatment on the success of assisted reproductive technology is lacking. To investigate the gut microbiota in fertile and infertile females, we conducted 16S metagenomic analysis of fecal samples. In total 18 fertile female subjects and 18 patients with infertility matched by age were recruited, and fecal samples were obtained to analyze the gut microbiome using 16S rRNA V3–V4 sequencing. The unweighted and weighted principal coordinate analyses showed a trend indicating microbial structural differences in β-diversity between these two groups. The abundance of the phylum Verrucomicrobia was higher in patients with infertility. At the genus level, a decrease in the abundance of the genera *Stenotrophomonas*, *Streptococcus*, and *Roseburia* and an increase in the abundance of the genera Unclassified [*Barnesiellaceae*] and *Phascolarctobacterium* was observed in patients with infertility. Twelve patients agreed to receive the combined therapy comprising embryo transfer by assisted reproductive technology and oral supplementation with partially hydrolyzed guar gum. The success of pregnancy by this combined therapy was 58.3% (7/12), and the failure was 41.7% (5/12). Predictive factors for pregnancy before treatment were characterized by a decrease in the abundance of *Paraprevotella* and *Blautia* and an increase in the abundance of *Bifidobacterium*. Predictive factors for pregnancy before treatment were characterized by a decrease in the abundance of *Paraprevotella* and *Blautia* and an increase tendency in the abundance of *Bifidobacterium*. In conclusion, the present study showed differences in the abundance of gut microbiota between fertile and infertile groups; moreover, partially hydrolyzed guar gum supplementation helped improve gut dysbiosis and the success of pregnancy in females with infertility.

## Introduction

In 2012, a national, regional, and global survey reported that the absolute number of couples affected by infertility increased from 42.0 million in 1990 to 48.5 million in 2010.^(1)^ Despite the advances in assisted reproductive technology (ART) in women as well as men, approximately 8–12% of the global population willing to conceive is unable to do so. Available evidence suggests that vaginal and uterine microbiota have a close relationship with the female infertility.^(2–4)^ In fact, microbiota analysis using the 16S rRNA amplicon sequencing of cervical swabs revealed significant differences regarding the relative read count of the *Gardnerella* genus between females diagnosed with infectious infertility and fertile controls.^(2)^

In addition, it has been proposed that dysbiosis of gut microbiota may be a potential pathogenic factor in the development of the polycystic ovary syndrome (PCOS), a widespread endocrine disease associated with high risk of infertility.^(5)^ Kelley *et al.*^(5)^ have suggested that the hyperandrogenemia observed in PCOS may consederably alter the gut microbiome independently of diet. Nishijima *et al.*^(6)^ have demonstrated that the gut microbiota of the Japanese population is considerably different from that of other populations. However, little is known about the differences in gut microbiota between women with infertility and fertile controls.

The diet of industrialized nations, including Japan, has experienced a decrease in fiber intake, which is well below the recommended daily range of 24 g per day for adults, and this deficit has been linked to several common diseases.^(7)^ The decrease in dietary fiber intake has been reported to induce dysbiosis with a decrease in the products derived through fermentation. Partially hydrolyzed guar gum (PHGG) is defined as a form of prebiotic dietary water-soluble fiber whose supplementation may modulate the gut microbiota in healthy adults and patients with diarrhea, constipation, and stress-related diseases.^(8–10)^

In the present study, we aimed to compare the gut microbiota in female patients with infertility with that of fertile subjects, and evaluate the effect of prebiotic PHGG supplementation on gut dysbiosis and the outcome of pregnancy in patients treated with ART.

## Materials and Methods

### Ethics statement

This study conformed to the code of ethics stated in the Declaration of Helsinki. The Medical Corporation Sankeikay Institutional Review Board approved the research protocol (permission No. 2017-27), and all participants provided written informed consent before enrollment. The protocol of microbiome analysis was also approved by the Ethics Committee of Kyoto Prefectural University of Medicine (permission No. ERB-C-534).

### Subjects and study protocol

In the first stage, 18 women with infertility and 18 fertile controls were prospectively selected from the HORAC Grand Front Osaka Clinic from the April 2017 to December 2018. Details of the individuals in this study are summarized in Table 1. There were no significant differences in age, body mass index, or diet style. In the second stage, 12 patients in the infertility group were given PHGG supplementation in addition to embryo transfer therapy. For easy ingestion, PHGG (Sunfiber^®^, 10 g/day; Taiyo Kagaku Co. Ltd., Yokkaichi, Japan) was dissolved in either food or a drink and administered to patients for 4 weeks. PHGG can be added without affecting the appearance or flavor of the diets.

### Sample collection and DNA extraction

Fecal samples, the size of a grain of rice, were collected using a feces collection kit (Techno Suruga Lab, Shizuoka, Japan). After vigorous mixing, the samples were stored at room temperature for 7 days until DNA extraction. Genomic DNA was isolated using the NucleoSpin Microbial DNA Kit (MACHEREY-NAGEL, Düren, Germany). Approximately 500 µl of the stored fecal samples were placed into microcentrifuge tubes containing 100 µl of elution buffer BE. The mixture was then placed into NucleoSpin Beads Tubes with Proteinase K, and subjected to mechanical bead-beating for 12 min at 30 Hz in the TissueLyzer LT (Qiagen, Hilden, Germany). The subsequent extraction procedure was performed according to the manufacturer’s instructions. Extracted DNA samples were purified using Agencourt AMPure XP (Beckman Coulter, Brea, CA).

### Sequencing of the 16S rRNA gene

Purified DNA samples were subjected to two step polymerase chain reactions (PCRs) to obtain sequence libraries. The first PCR was performed to amplify using the 16S rRNA gene using the (V3–V4) Metagenomic Library Construction Kit for NGS (Takara Bio Inc., Kusatsu, Japan) with primer pairs 341F (5'-TCGTCGGCAGCGTCAGAT GTGTATAAGAGACAGCCTACGGGNGGCWGCAG-3') and 806R (5'-GTCTCGTGGGCTCGGAGATGTGTATAAGAGAC AGGGACTACHVGGGTWTCTAAT-3') corresponding to the V3–V4 region of the 16S rRNA gene. The second PCR was conducted to add the index sequences for the Illumina sequencer with barcode sequence using the Nextera Index kit (Illumina, San Diego, CA). The prepared libraries were subjected to sequencing paired-end 250 bases using the MiSeq Reagent v3 600-cycles kit on the MiSeq (Illumina) at the Biomedical Center, Takara Bio Inc.

### Microbiome analysis

The processing of sequence data, including the quality filter of sequence reads, chimera check, and operational taxonomic unit (OTU) definition (97% identity), was performed using the QIIME ver. 1.9,^(11)^ USEARCH ver. 9.2.64, UCHIME ver. 4.2.40^(12,13)^ and VSEARCH ver. 2.4.3.^(14)^

To assign OTUs at the phylum to genus level, each OTU was aligned to the GreenGenes database (ver. 13.8) with the RDP classifier (ver. 2.2) algorithm by QIIME (ver. 1.9). Singletons were removed from the analysis. Statistical differences (*p*<0.05) for the relative abundance of bacterial phyla and genera between groups were evaluated using Welch’s unpaired *t* tests.

The observed species, Chao1 and Shannon phylogenetic diversity indices were calculated uisng the R “phyloseq” package and statistically analyzed using a Wilcoxon rank-sum test. β-Diversity was estimated using the UniFrac metric to calculate the distances between the samples that were visualized using the principal coordinate analysis (PCoA) and statistically analyzed using permutational multivariate analysis of variance (PERMANOVA). The figures were generated using QIIME software (ver. 1.9).

## Results

### Comparison of the gut microbiota between control subjects and patients with infertility

**Fig. 1:**
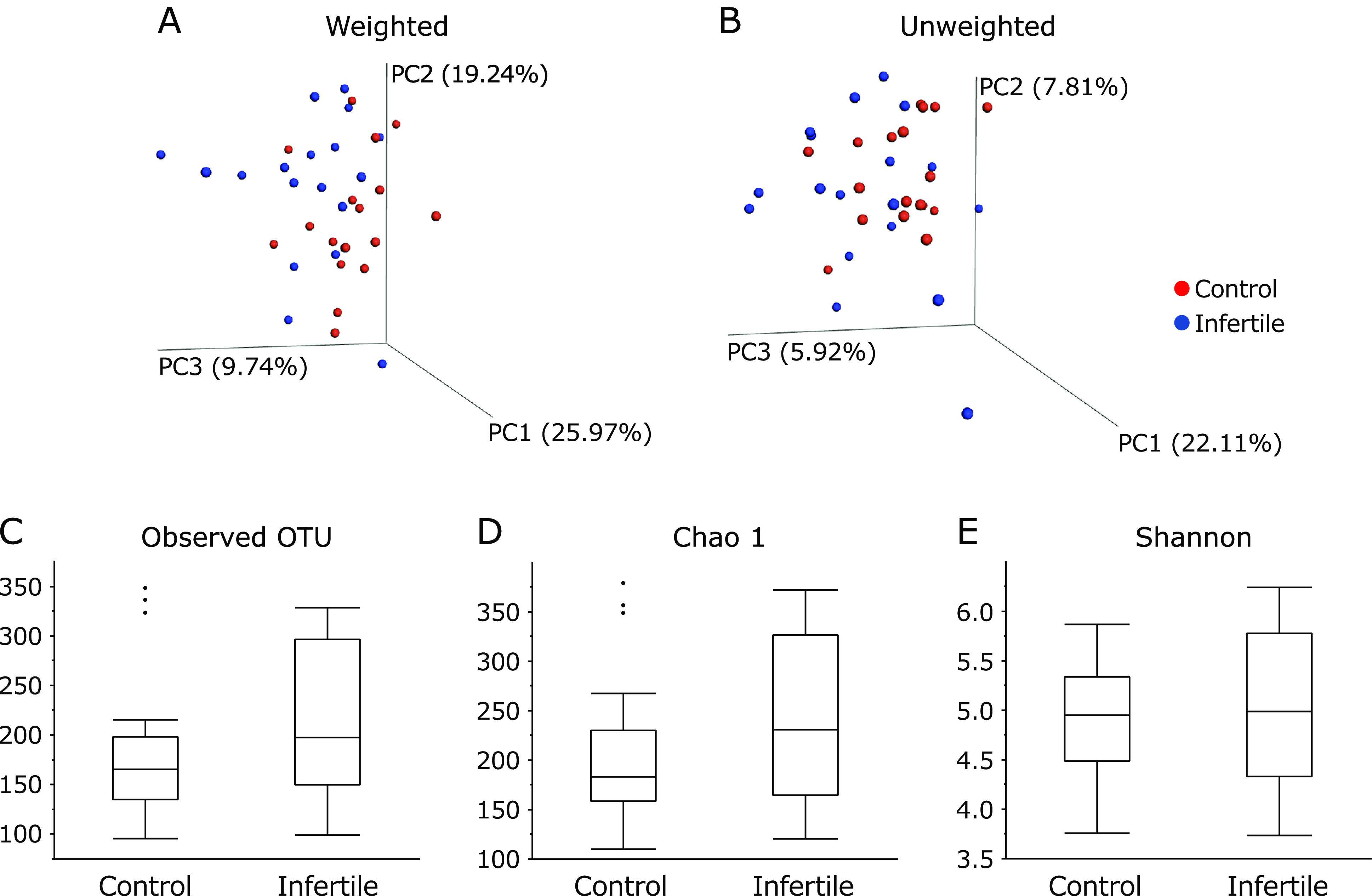
Principal coordinate analysis (PCoA) plots of control subjects vs patients with infertility and the α-diversity indices between the two groups. Distances were calculated with weighted (A) and unweighted (B) UniFrac distances. (C) observed species, (D) the Chao 1 index (OUT richness estimation), (E) the Shannon index (OUT evenness estimation).

A total of 734 OTUs were detected in this study. Initially, the overall structure of the gut microbiome between control subjects and patients with infertility was calculated using β-diversity indices for unweighted and weighted UniFrac distances (Fig. 1). PCoA revealed a trend indicating microbial structural differences between control subjects and patients with infertility in unweighted (PERMANOVA *p* = 0.06) and weighted (PERMANOVA *p* = 0.11) UniFrac distances. Subsequently, we compared α-diversity between control subjects and patients with infertility using different indices such as the observed species, the Chao 1 index (OTU richness estimation) and the Shannon index (OTU evenness estimation). (Fig. 1) Unfortunately, there was no statistically significant difference in α-diversity between these two groups, though the observed species and the Chao 1 index showed an increasing trend in the patients in the infertility group.

**Fig. 2:**
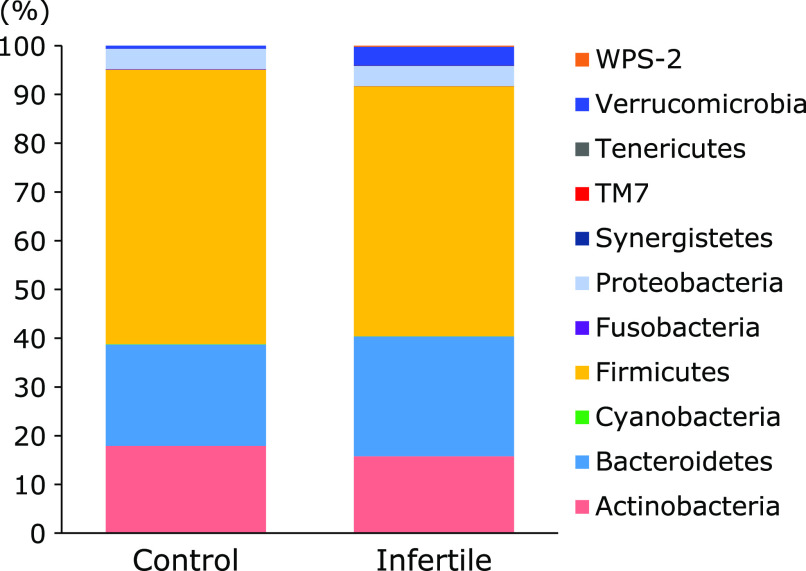
Comparative analyses of the taxonomic composition of the microbial community at the phylum level. Each component of the cumulative bar chart indicates a phylum.

Differences in the gut microbial structure were taxonomically evaluated at the phylum level (Fig. 2). No significant difference was found in the abundance of the phyla Actinobacteria, Firmicutes, Bacteroidetes, and Proteobacteria between control subjects and patients with infertility. In contrast, the abundance of the phylum Verrucomicrobia was considerably higher in the infertile patients group than in the control group, although the difference was not statistically significant (*p* = 0.13).

**Fig. 3:**
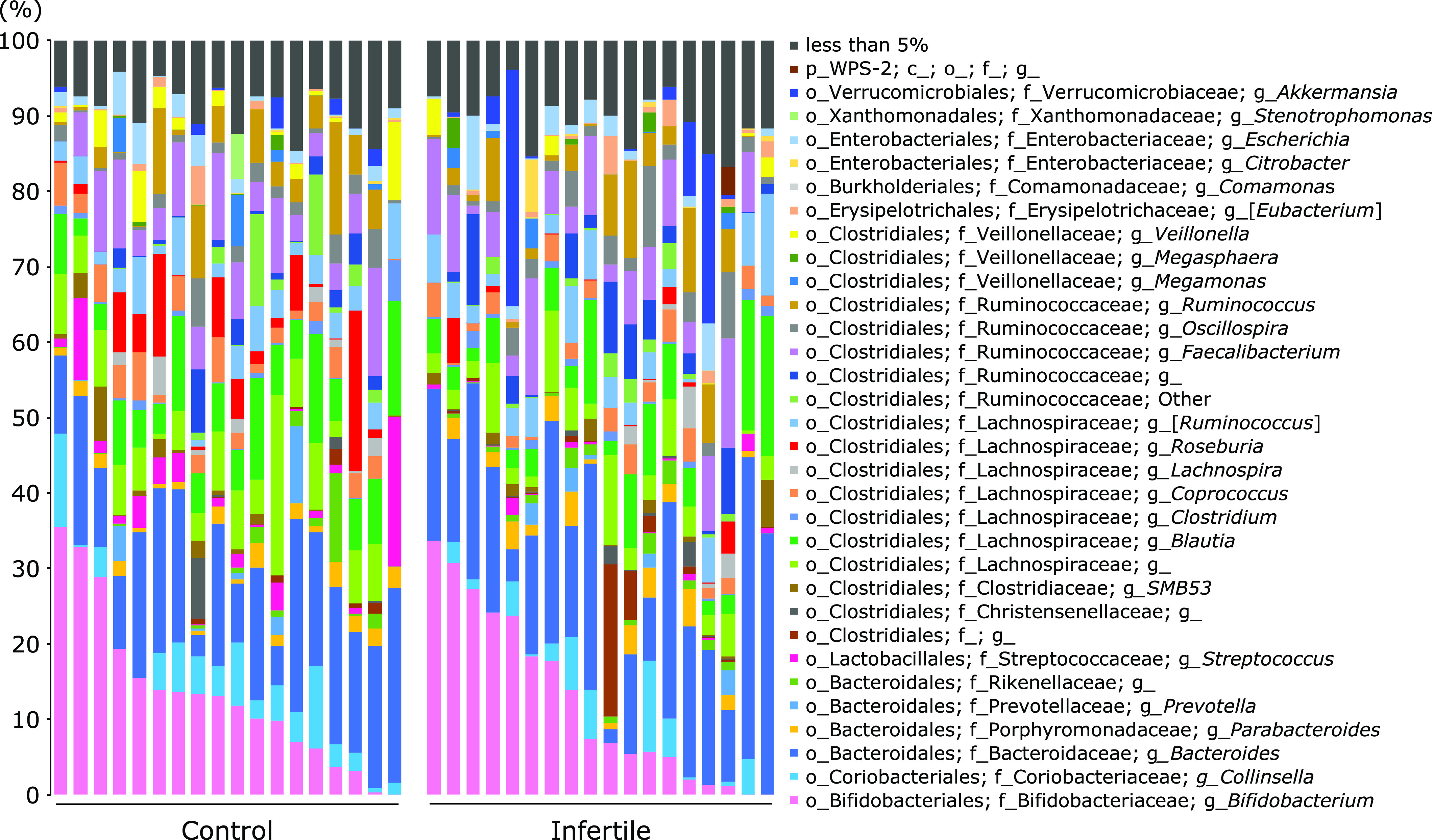
Taxonomic composition of the microbial community at the genus level for each control subject and patients with infertility.

**Fig. 4:**
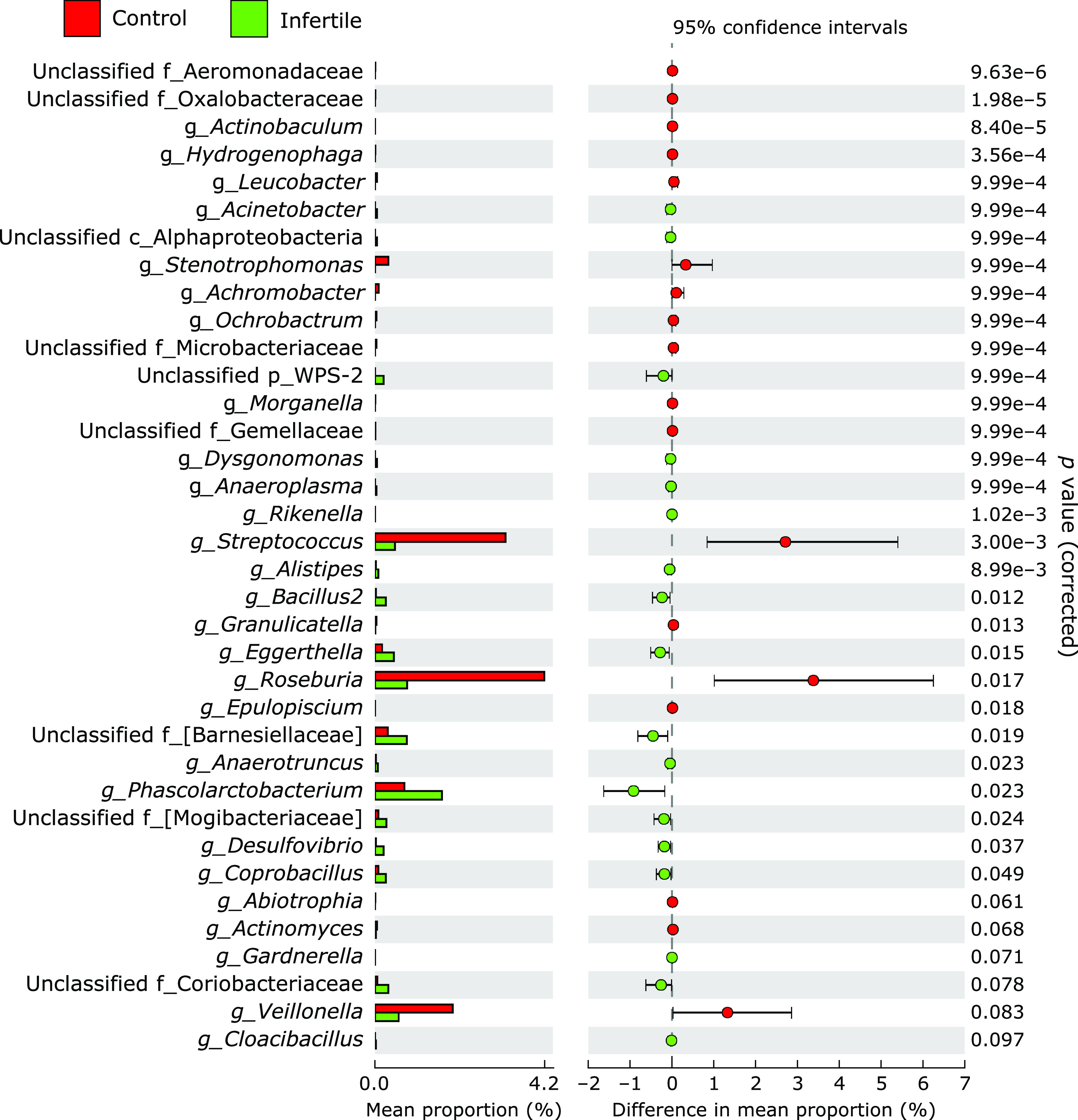
Comparative analysis of the taxonomic composition of the microbial community at the genus level between control subjects and patients with infertility. Representative genera with significant and trend to significant differences between groups are presented.

The taxonomic composition of the microbial community was evaluated at the genus level. As shown in Fig. 3, the abundance of *Bifidobacterium* varied greatly from individual to individual, ranging from 0% to a maximum of 35%. The comparison of the microbial composition between control subjects and patients with infertility showed a significant decrease or increase in the abundance of several genera (Fig. 4). These genera were characterized by a decrease in the abundance of the genera *Stenotrophomonas*, (*p*<0.01), *Streptococcus* (*p*<0.01) and *Roseburia* (*p*<0.05) and an increase in the abundance of the genera Unclassified [*Barnesiellaceae*] (*p*<0.05) and *Phascolarctobacterium* (*p*<0.05) in patients with infertility.

### Effect of prebiotic PHGG supplementation on gut dysbiosis

**Fig. 5:**
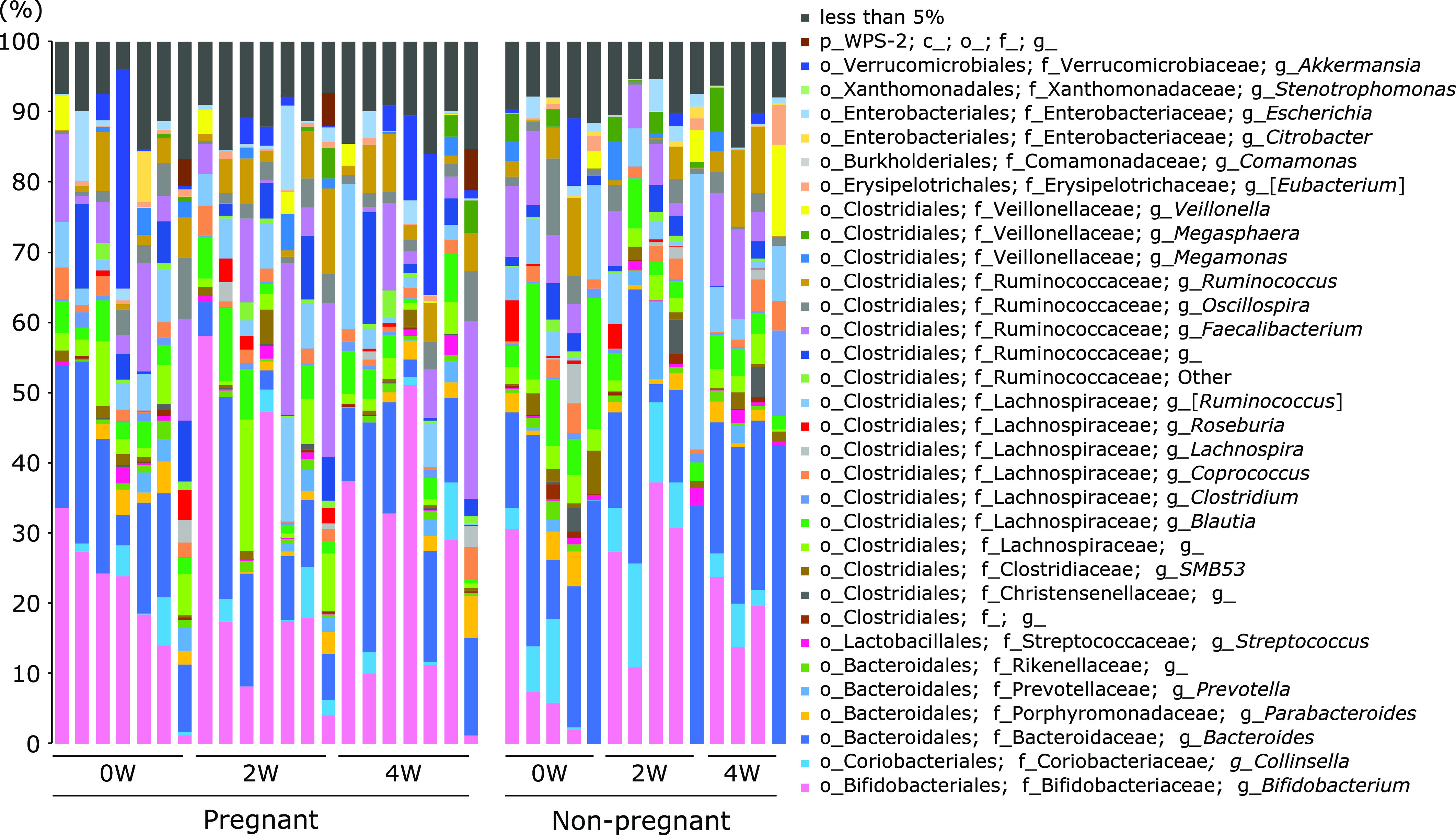
Changes in taxonomic composition of the microbial community at the genus level in the pregnant and the non-pregnant groups during treatment with partially hydrolyzed guar gum.

**Fig. 6:**
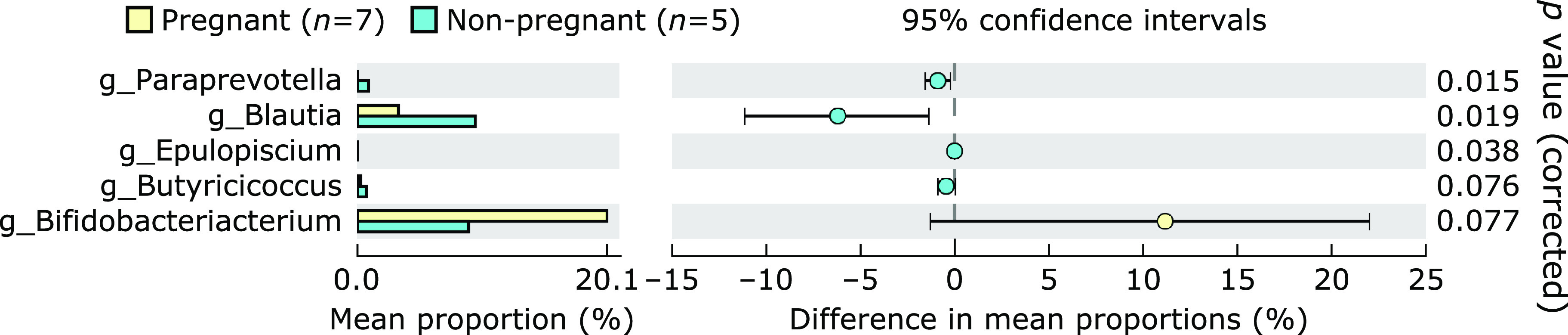
Comparative analysis of the taxonomic composition of the microbial community at the genus level between the pregnant and the non-pregnant groups before treatment with partially hydrolyzed guar gum. Representative genera with significant and trend to significant differences between groups are presented.

Twelve out of 18 patients with infertility agreed to receive the combined therapy with embryo transfer by ART and additional oral PHGG supplementation. The obtained ongoing pregnancy by the combined therapy was 58.3% (7/12), and the failure of pregnancy was 41.7% (5/12). Figure 5 shows changes in the taxonomic composition of the microbial community at the genus level in the pregnant and the non-pregnant groups during PHGG treatment. The abundance of the genus *Bifidobacterium* tended to increase 2 and 4 weeks after the PHGG treatment in the pregnant group, and the abundance of the genus *Bacteroides* tended to increase in the non-pregnant group. To confirm the predictive genera for the success of pregnancy, the taxonomic composition of the microbial community was compared between groups at the genus level before the PHGG treatment. As shown in Fig. 6, the predictive factors for pregnancy were characterized by a decrease in the abundance of *Paraprevotella* and *Blautia* and an increase tendency in the abundance of *Bifidobacterium*.

## Discussion

In the present study, we used fecal DNA samples obtained from fertile control females and patients with infertility and performed a comparative study using data obtained the sequencing from 16S rRNA genesequencing. We described the differences in the relative abundance of gut microbiota between controls and females with infertility. Initially, using the unweighted and weighted UniFrac distances, we compared the overall microbial structure between these two groups. Importantly, as presented in Fig. 1, the unweighted and weighted PCoAs showed a trend indicating microbial structural differences between these two groups. The α-diversity indices between the two groups revealed no significant differences, although there appeared to be a higher distribution of diversity among patients with infertility than control subjects. Second, the differences in the gut microbial structure were taxonomically evaluated at the phylum and genus levels. Although there was no significant difference in the abundance of the phyla Actinobacteria, Firmicutes, Bacteroidetes, and Proteobacteria between these two groups, the abundance of the phylum Verrucomicrobia tended to be higher in patients with infertility. One of the most striking results was a decrease in the abundance of the genera *Stenotrophomonas*, *Streptococcus* and *Roseburia*, and an increase in the abundance of the genera Unclassified [*Barnesiellaceae*] and *Phascolarctobacterium* in patients with infertility compared with those in control subjects.

Recent findings from the Human Microbiome Project demonstrate that many different species of *Lactobacillus* are present in the vaginal tract.^(15)^ Moreno *et al.*^(3)^ have showed that the presence of a non-*Lactobacillus*-dominated microbiota in a receptive endometrium is associated with significant decreases in implantation, pregnancy, ongoing pregnancy, and live birth rates. Moreover, the microbiota composition in the ovarian follicular fluid is associated with reproductive outcomes after *in vitro* fertilization.^(16)^ However, only few studies have reported differences in gut microbiome profiles between fertile and infertile females. To our knowledge, this is the first report to reveal the differences in gut microbiota between fertile females and females with infertility, which showed a trend indicating an increase in the abundance of the phylum Verrucomicrobia in patients with infertility. Verrucomicrobia was represented by a single genus, *Akkermansia*, that has only one species described, *A. muciniphila*. This species is thought to have a potentially anti-inflammatory effect in humans and induces regulatory T cells. A study has reported that *A. muciniphila* affects glucose and lipid metabolism and intestinal immunity and that certain food ingredients, such as polyphenols, may increase the abundance of *A. muciniphila* in the gut.^(17)^ In contrast to these beneficial effects of *A. muciniphila*, the present study showed a trend to toward an increase in the abundance of Verrucomicrobia in patients with infertility compared with fertile subjects. The clear reasons for the increase in this bacterium could not be explained; thus, large-scale controlled trials are needed in the future.

A registry study of the Japan Society of Obstetrics & Gynecology (JSOG) has reported that the pregnancy rate for the ART treatment was 36.2% for patients aged 37 years, similar to the mean age of the present study,^(18)^ indicating that the result of this study (58.3%, 7/12), was relatively high. However, this study is a single arm intervention study with PHGG; irrespective, prospective comparative studies are needed in the future. Next, to predict the outcome of the PHGG + ART combination treatment, the abundances of gut microbiota before treatment were compared between the successful and failed pregnancy groups. We confirmed that the predictive factors for pregnancy were characterized by a decrease in the abundance of *Paraprevotella* and *Blautia*, and an increase tendency in the abundance of *Bifidobacterium*. As shown in Fig. 6, *Blautia* and *Bifidobacterium* were the predominant fecal bacteria in this study. Both these bacteria have been reported to be involved in the fermentation process to produce short-chain fatty acids and to affect the intestinal environment. Although further investigation is required to determine whether changes in the relative abundance of *Blautia* and *Bifidobacterium* in the gut microbiota affect risk factors related to the pregnancy rate following ART, these bacteria may have the potential to maintain or improve reproductive processes and could potentially be a new target or index for the success of ART.

In the present study, we also evaluated the changes in the taxonomic composition of the microbial community at the genus level during the PHGG treatment. As shown in Fig. 5, the abundance of *Bifidobacterium* increased 2 weeks after PHGG administration and stabilized after 4 weeks in the successful pregnancy group. In contrast, the increased abundance of *Bifidobacterium* 2 weeks after PHGG treatment decreased 4 weeks after treatment in the failed pregnancy group. These data suggest the possibility that the response of gut microbiota to the dietary fiber treatment may affect the success of pregnancy. In agreement with our findings, previous studies have also reported discordant results when investigating the gut microbiota of subjects treated with PHGG. Okubo *et al.*^(19)^ were the first to report that PHGG increased the fecal *Bifidobacterium* spp. after 2 weeks of intake in healthy male volunteers. Yasukawa *et al.*^(9)^ recently performed a randomized, double-blind, placebo-controlled, parallel trial in healthy volunteers to investigate the effect of PHGG on the tendency to defecate loose stools, and demonstrated that PHGG increases the abundance of *Bifidobacterium* and decreases the abundance of *Bacteroides*. *Bifidobacterium* is a genus that produces lactic and acetic acids via the fermentation of dietary fibers. Finally, it should be noted that the abundance of *Bifidobacterium* in the feces varies greatly between individuals. As highlighted in previous reports targeting the Japanese population, *Bifidobacterium* is extremely important for maintaining the intestinal environment in the large intestine. These results suggest that the effects of the gut microbiota, especially *Bifidobacterium*, on pregnancy should be analyzed for each person considering the differences in the individual gut microbiota.

In summary, the present study showed the differences in the abundance of gut microbiota between fertile and infertile female groups, and that PHGG supplementation helped improve gut dysbiosis and the success of pregnancy in females with infertility. Further studies are needed to elucidate the mechanisms involved in this phenomenon, and a clinical study using a large number of samples is highly recommended as it will provide more solid evidence and confirm our findings.

## Author Contributions

SK, HO, YM, KH, and YM enrolled the patients and control subjects and collected fecal samples. YN, TT, KM, RI and AA conducted the analyses. SK, YN and RI wrote the manuscript. All authors have read and approved the final manuscript.

## Figures and Tables

**Table 1:** Baseline characteristics of enrolled subjects in the first stage

	Fertile control	Females with infertility	*p*
*n*	18	18	
Age (years)	34.83 ± 2.64	36.39 ± 2.77	ns
Body mass index (kg/m2)	20.78 ± 2.39	21.41 ± 3.34	ns
Duration of ART* (month)	—	42.8 ± 24.1	
Embryo transfer therapy (*n*)	—	12	
Cesarean section (*n*)	2	2	ns
Diets**(*n*)			
High carbohydrate	6	4	ns
High protein	0	1	ns
High lipid	0	0	ns

*Assisted reproductive technology therapy, **High carbohydrate diet defined as a diet that consumes at least 66% of total energy from carbohydrates, high protein ≥21% from animal protein, and high lipid ≥31% from lipid.
